# Supplementary material for: A yeast phenomic model for the influence of Warburg metabolism on genetic buffering of doxorubicin
Source: Cancer Metab. 2019 Oct 23;7:9. doi: 10.1186/s40170-019-0201-3 (PMC6806529; doi:10.1186/s40170-019-0201-3)
Supplement: Supplementary file 9 — Additional file 9. GO term-specific heatmaps for REMc/GTF-enriched clusters. GO term-specific heatmaps for significant GO process terms were generated as described in methods and Figs. 3 and 4. Any related child terms are presented in subsequent pages of the parent file name. GO terms with more than 100 children, with 2 or fewer genes annotated to the term, or a file size over 300KB are not shown. All heatmaps are generated with the same layout (see Figs. 3 and 4). [file 40170_2019_201_MOESM9_ESM.bz2 › Additional_File9_GOTermHeatmaps/Additional_File9_GOTermHeatmap/positive_regulation_of_translation.pdf]

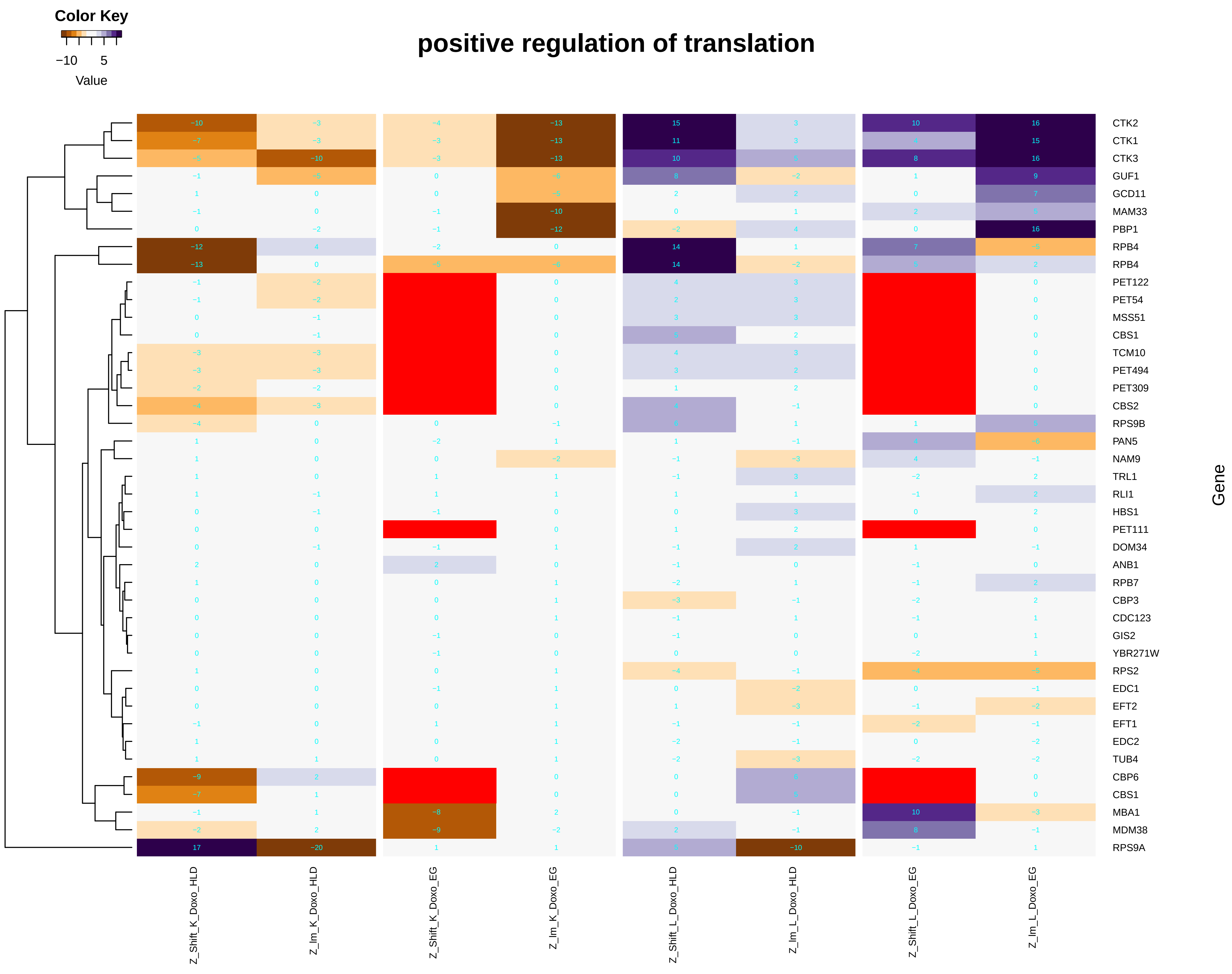
positive regulation of translation
Color Key
−10 5
Value
CTK2
CTK1
CTK3
GUF1
GCD11
MAM33
PBP1
RPB4
RPB4
PET122
PET54
MSS51
CBS1
TCM10
PET494
PET309
CBS2
RPS9B
PAN5
NAM9
TRL1
RLI1
HBS1
PET111
DOM34
ANB1
RPB7
CBP3
CDC123
GIS2
YBR271W
RPS2
EDC1
EFT2
EFT1
EDC2
TUB4
CBP6
CBS1
MBA1
MDM38
RPS9A
Gene
Z_Shift_K_Doxo_HLD
Z_Im_K_Doxo_HLD
Z_Shift_K_Doxo_EG
Z_Im_K_Doxo_EG
Z_Shift_L_Doxo_HLD
Z_Im_L_Doxo_HLD
Z_Shift_L_Doxo_EG
Z_Im_L_Doxo_EG

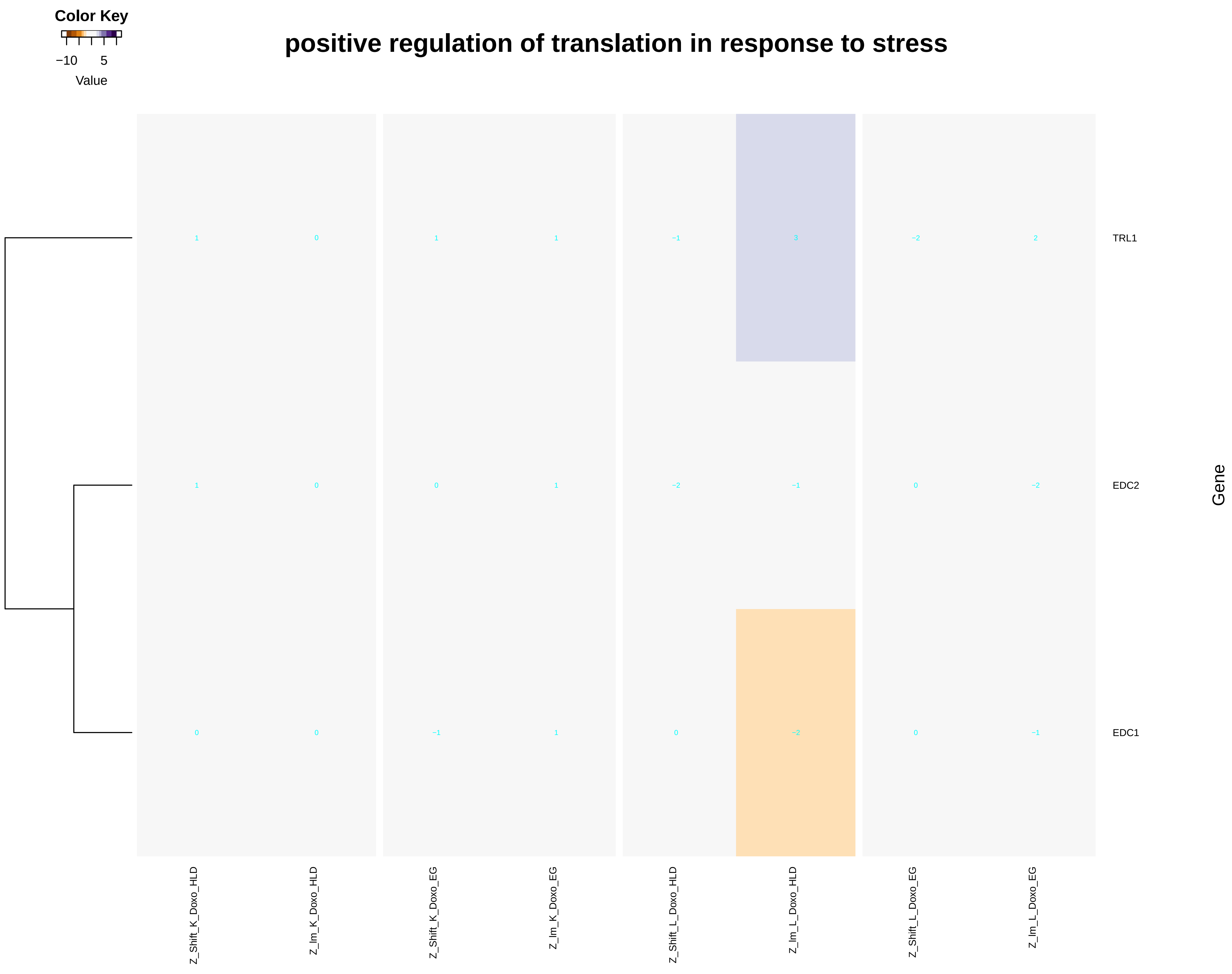

Color Key
−10
5
Value
positive regulation of translation in response to stress
1
0
1
1
−1
3
−2
2
TRL1
1
0
0
1
−2
−1
0
−2
EDC2
0
0
−1
1
0
−2
0
−1
EDC1
Gene
Z_Shift_K_Doxo_HLD
Z_Im_K_Doxo_HLD
Z_Shift_K_Doxo_EG
Z_Im_K_Doxo_EG
Z_Shift_L_Doxo_HLD
Z_Im_L_Doxo_HLD
Z_Shift_L_Doxo_EG
Z_Im_L_Doxo_EG

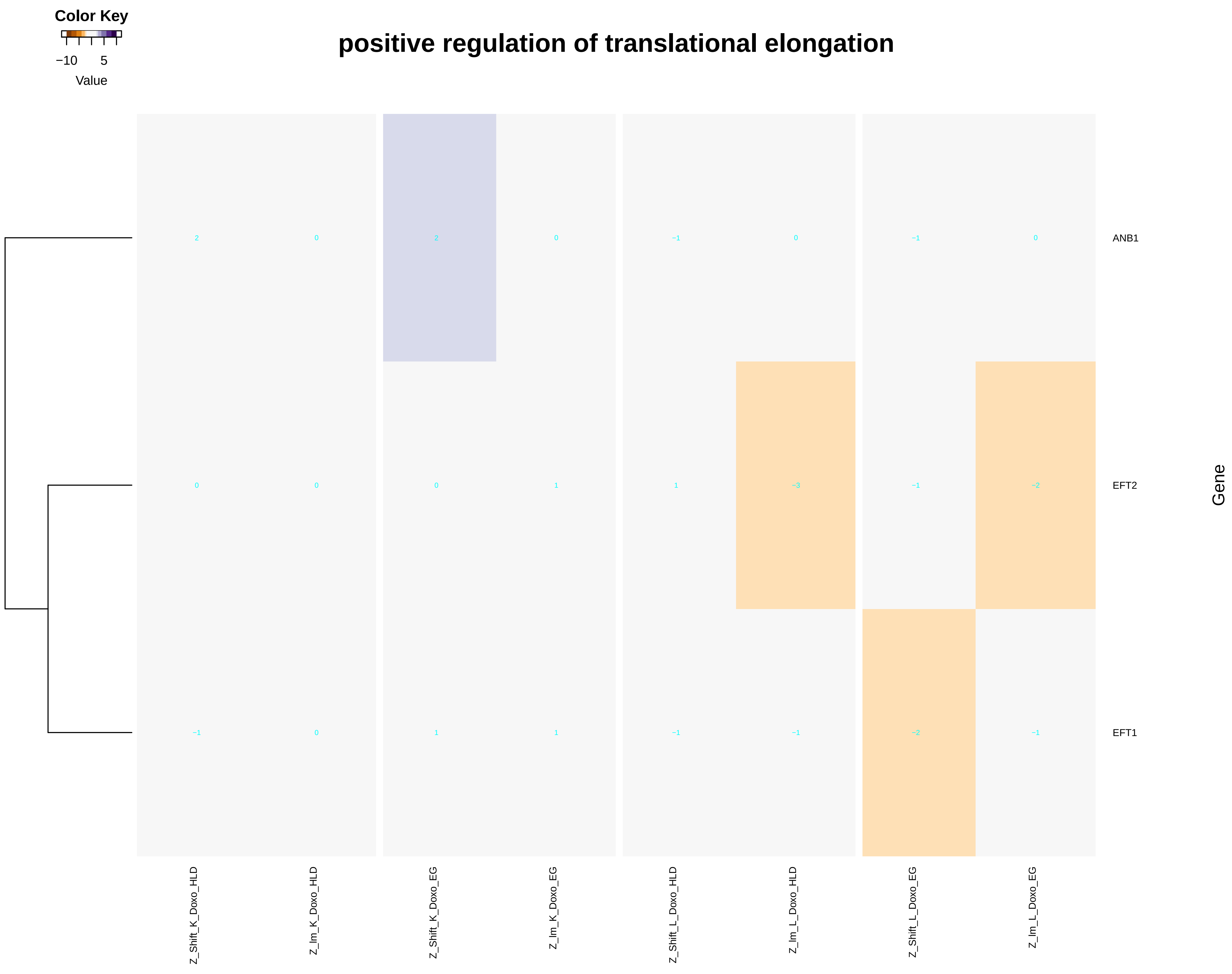
Color Key
−10
5
Value
positive regulation of translational elongation
2
0
2
0
−1
0
−1
0
ANB1
0
0
0
1
1
−3
−1
−2
EFT2
−1
0
1
1
−1
−1
−2
−1
EFT1
Gene
Z_Shift_K_Doxo_HLD
Z_Im_K_Doxo_HLD
Z_Shift_K_Doxo_EG
Z_Im_K_Doxo_EG
Z_Shift_L_Doxo_HLD
Z_Im_L_Doxo_HLD
Z_Shift_L_Doxo_EG
Z_Im_L_Doxo_EG

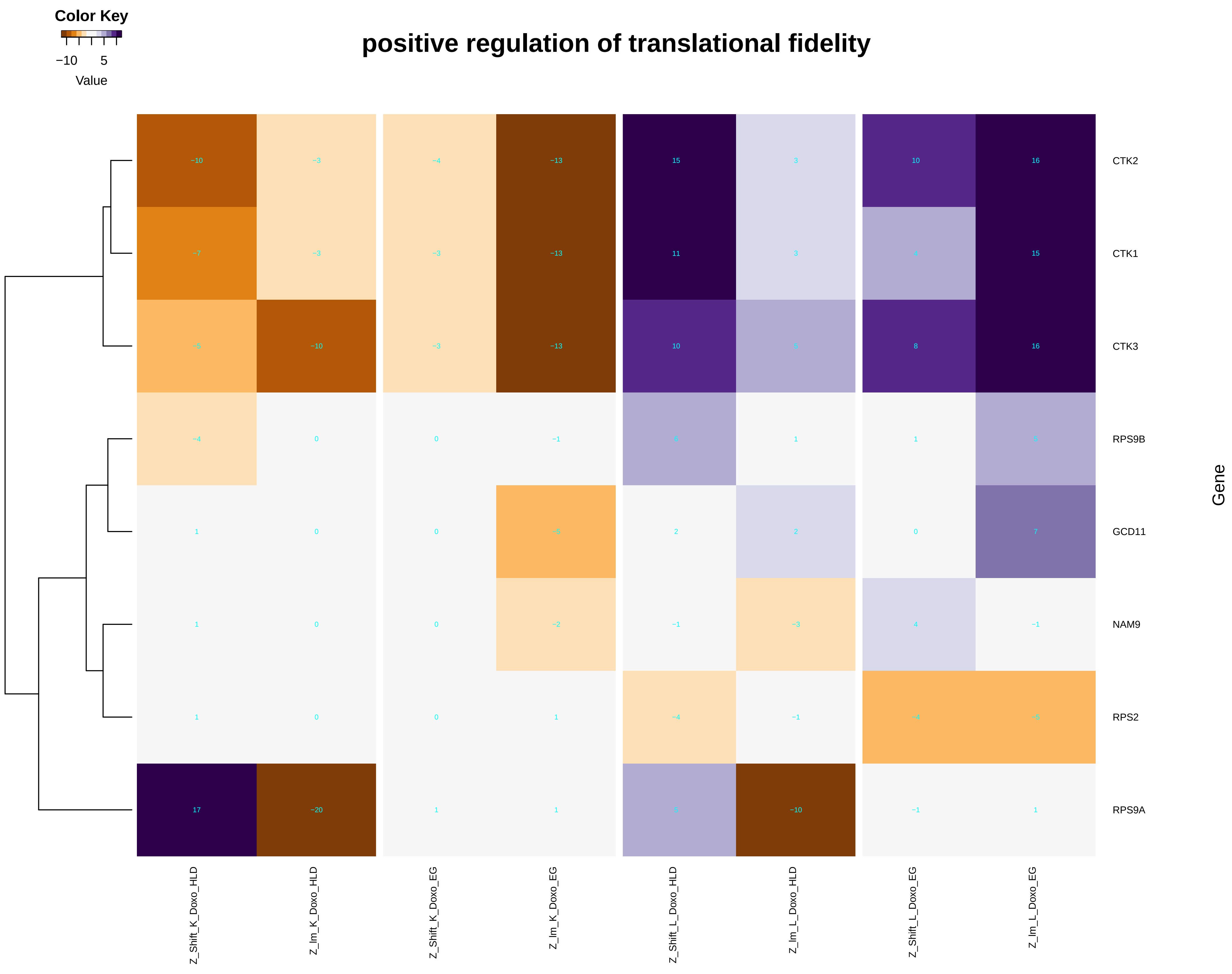
Color Key
−10
5
Value
positive regulation of translational fidelity
CTK2
CTK1
CTK3
RPS9B
GCD11
NAM9
RPS2
RPS9A
Gene
Z_Shift_K_Doxo_HLD
Z_Im_K_Doxo_HLD
Z_Shift_K_Doxo_EG
Z_Im_K_Doxo_EG
Z_Shift_L_Doxo_HLD
Z_Im_L_Doxo_HLD
Z_Shift_L_Doxo_EG
Z_Im_L_Doxo_EG

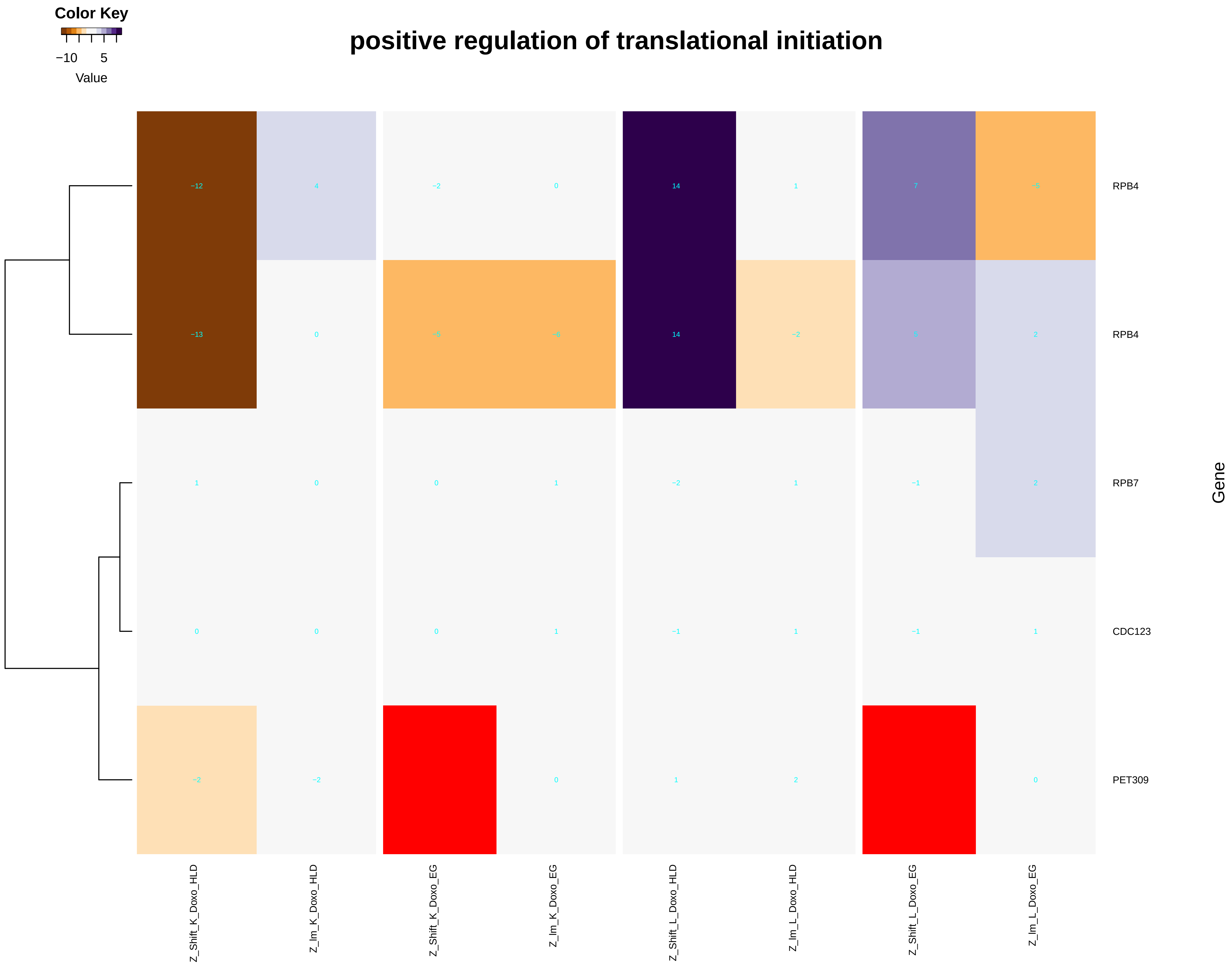
Color Key
−10
5
Value
positive regulation of translational initiation
RPB4
RPB4
RPB7
CDC123
PET309
Gene
Z_Shift_K_Doxo_HLD
Z_Im_K_Doxo_HLD
Z_Shift_K_Doxo_EG
Z_Im_K_Doxo_EG
Z_Shift_L_Doxo_HLD
Z_Im_L_Doxo_HLD
Z_Shift_L_Doxo_EG
Z_Im_L_Doxo_EG

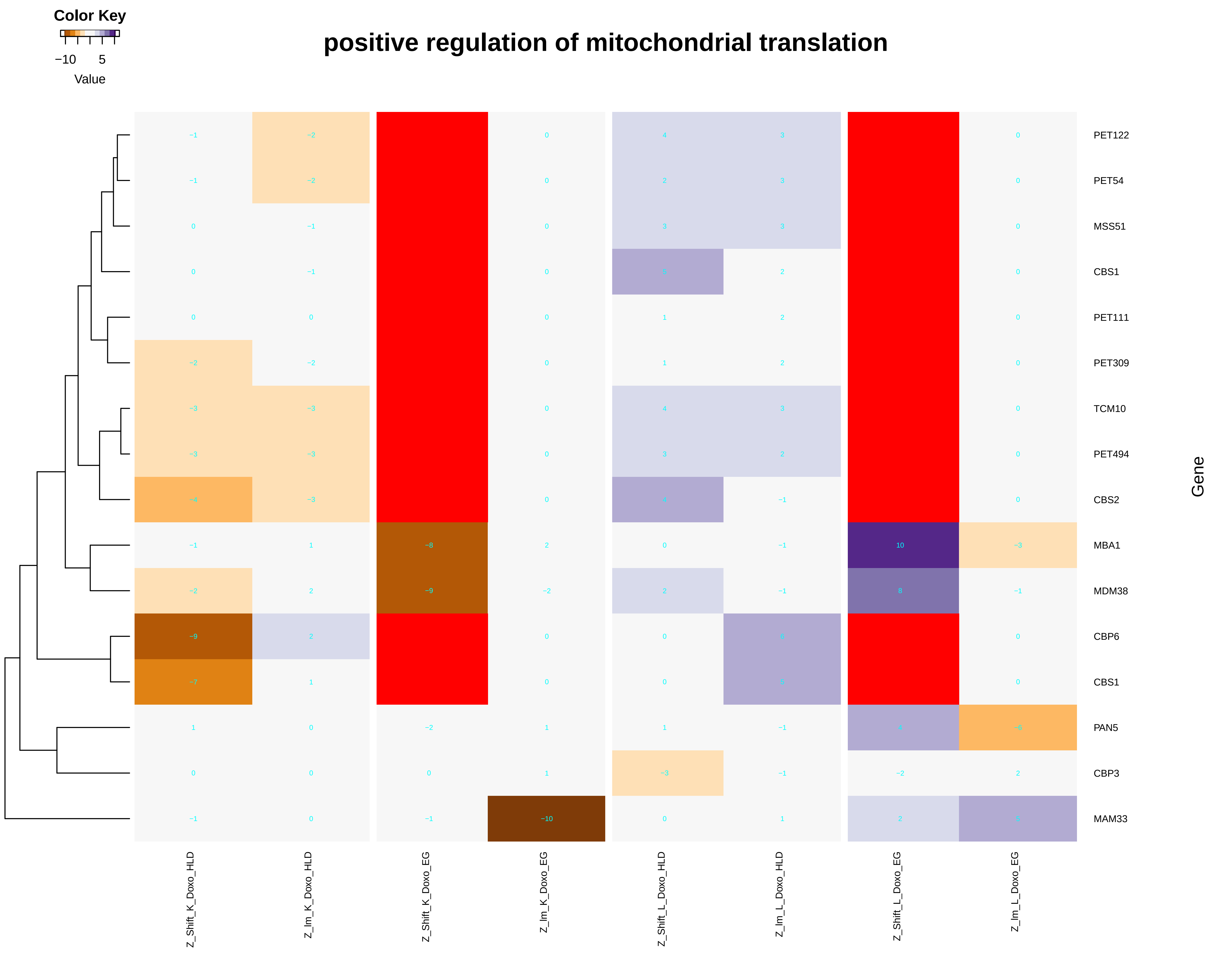

Color Key
−10 5
Value
positive regulation of mitochondrial translation
PET122
PET54
MSS51
CBS1
PET111
PET309
TCM10
PET494
CBS2
MBA1
MDM38
CBP6
CBS1
PAN5
CBP3
MAM33
Gene
Z_Shift_K_Doxo_HLD
Z_Im_K_Doxo_HLD
Z_Shift_K_Doxo_EG
Z_Im_K_Doxo_EG
Z_Shift_L_Doxo_HLD
Z_Im_L_Doxo_HLD
Z_Shift_L_Doxo_EG
Z_Im_L_Doxo_EG
